# Pre-pandemic physical activity as a predictor of infection and mortality associated with COVID-19: Evidence from the National Health Insurance Service

**DOI:** 10.3389/fpubh.2023.1072198

**Published:** 2023-02-14

**Authors:** Saengryeol Park, Hyeseong Kim, So-Youn Park, In-Hwan Oh

**Affiliations:** ^1^Department of Physical Education, College of Education, Chonnam National University, Gwangju, Republic of Korea; ^2^Department of Medical Education and Humanities, School of Medicine, Kyung Hee University, Seoul, Republic of Korea; ^3^Preventive Medicine (AgeTech-Service Convergence Major), College of Medicine, Kyung Hee University, Seoul, Republic of Korea

**Keywords:** physical activity, COVID-19, quarantine, lifestyle, COVID-19-associated mortality

## Abstract

**Introduction:**

During the coronavirus disease 2019 (COVID-19) pandemic, many populations have experienced reduced physical activity (PA) levels, weight gain, and increased anxiety and depression. However, according to a previous study, engaging in PA has a positive effect on damages caused by COVID-19. Therefore, this study aimed to investigate the association between PA and COVID-19 using the National Health Insurance Sharing Service Database in South Korea.

**Methods:**

Logistic regression analysis was used to analyze the association of PA with COVID-19 and mortality. The analysis was adjusted for body mass index, sex, age, insurance type, comorbidity, and region of residence at baseline. Disability and lifestyle (weight, smoking, and drinking status) were adjusted consecutively.

**Results:**

The results indicated that engaging in insufficient PA as per the WHO guidelines predicts a higher risk of COVID-19 when controlling for personal characteristics, comorbidity, lifestyle, disability, and mortality.

**Discussion:**

This study revealed the need to engage in PA and manage weight to reduce the risk of infection and mortality associated with COVID-19. Because engaging in PA is an important component of weight management and can help restore physical and mental health after the COVID-19 pandemic, it should be emphasized as a pillar of recovery after COVID-19.

## 1. Introduction

The novel coronavirus disease 2019 (COVID-19) has been a prominent global issue since its emergence in 2019. It is an infectious respiratory disease with mild-to-severe symptoms, which may include fever, cough, loss of taste or smell, and diarrhea (1, 2). However, in severe cases, chest pain, loss of speech, loss of mobility, and confusion may occur. COVID-19 is rapidly spread by respiratory droplets released during coughing, sneezing, speaking, singing, or breathing by the infected individual (3). Thus, the rapid spread of COVID-19 has caused an unprecedented number of cases and deaths. As of August 2022, there were over 593,236,266 confirmed cases and about 6,448,504 deaths worldwide (4). In South Korea, particularly, the fatality rate of older adults over 80 years old was 2.35% (5).

Recent studies show that COVID-19 has impacted mental health. There is evidence of increased severity of depression compared to those before pandemic (6, 7). It seems that quarantine throughout the COVID-19 pandemic negatively impacted the mental health of previously unaffected individuals; for example, the anxiety and depression of people whose family, colleagues, classmates, or neighbors were affected by quarantine were increased (8). In addition, the risks of anxiety, depression, stress, and sleep disorders in COVID-19 patients were increased (9). Post-traumatic stress symptoms were occasionally experienced after infection, but results concerning physical health were limited (10). However, it is unclear how factors relating to lifestyles are linked to the prognosis of COVID-19.

Engaging in physical activity (PA) has played an important role in improving psychological and physical health. It was found that engaging in regular moderate-to-vigorous PA (MVPA) is associated with reducing anxiety and negative self-perceptions, as well as improving physical health (11). In addition, engaging in MVPA is associated with losing and managing body weight that may predispose individuals to several types of chronic diseases, such as obesity and high blood pressure (12). However, a recent study shows that people spend more time engaging in sedentary behavior and less time engaging in PA than before the pandemic (13). Individuals engaging in PA is found to be more associated with lower risk of COVID-19 and mortality than those who do not meet the recommended PA level (150 min of MVPA at least once a week) (14, 15), although these results did not consider other factors of health such as personal characteristics, comorbidity, and disability level.

Thus, we aimed to examine the associations of MVPA with COVID-19 and mortality. To produce more robust study results, we tried to include controlling variables beyond MVPA, using nationally representative data.

## 2. Methods

### 2.1. Database

The National Health Insurance Service (NHIS) of South Korea is a social insurance system for the entire nation, and registration is compulsory; approximately 97% of the Korean population is currently registered (16). The NHIS assists people with scheduling medical checkups every 2 years and records their results automatically. These data include various information such as demographic information, payment specification, consultation statement, diagnosis statements, and prescriptions. With these records they developed the National Health Insurance Sharing Service for researcher to support various studies providing sample cohort DB, customized cohort DB, health screening cohort, etc.

This study used NHIS-COVID19 DB that included 4,363 adult COVID-19 patients in South Korea between January 1, 2020, and July 14, 2020, who had medical records between 2015 and 2018, the most recent data before the pandemic. We selected 67,125 adults for the control group in NHIS DB who also had medical checkup data. We used the most recent records in our study; the final data included COVID-19 status, demographic information, comorbidity, disability status, and lifestyle, including PA and body mass index (BMI). This study conformed to the Guidelines on De-identification of Personal Data of Korea and was approved by the Kyung Hee University's Institutional Review Board (IRB No. KHSIRB20-301[EA]) as a review exemption study. Thus, the requirement for informed consent was waived.

### 2.2. Variables

#### 2.2.1. PA

PA was measured using a self-report questionnaire from NHIS. Moderate PA (MPA) was measured with the following question: “During the last week, how many times a week and for how many hours a day did you engage in physical activity at a moderate level for more than 10 min (e.g., fast walking, doubles tennis, riding bicycle, cleaning)?” Vigorous PA (VPA) was assessed with the following question: “During the last week, how many days a week and for how many hours a day did you engage in physical activity at a vigorous level for more than 10 min (e.g., running, aerobic, fast riding a bicycle)?” PA was categorized into two groups according to PA guidelines: 150 min of MVPA at least once a week (1 min of VPA = 2 min of MPA). The items have been widely used in the literature (17).

#### 2.2.2. BMI

BMI is a simple obesity indicator calculated as weight/square of height (kg/m^2^). In this study, BMI was categorized into four groups according to the World Health Organization (WHO) BMI classification (underweight = BMI <18.5, normal range = 18.5 ≤ BMI <25, overweight = 25 ≤ BMI <30, obese = 30 ≤ BMI).

#### 2.2.3. Covariates

We adjusted for sex, age, region of residence, economic status, the number of comorbidities, disability, smoking status, drinking status, and weight, which are reportedly associated with COVID-19 (18, 19). Residence was categorized into the five regions (Seoul, Daegu, Gyeonggi, Gyeong-buk, and other) of South Korea (from January 1, 2020 to August 14, 2020) with the most confirmed COVID-19 cases.

Economic status was measured using health insurance premiums. Health insurance premiums are categorized into five quintiles. In South Korea, every person must pay part of their income as an insurance premium. Thus, a higher quintile indicates a higher economic status. Basic livelihood security recipients were included in the medical aid.

Comorbidity refers to an underlying condition (e.g., diabetes, hypertension) that may cause and affect other diseases. In this study, the number of comorbidities was investigated with the question: “Among the following diseases, which diseases have you been diagnosed with, or have you been treated for?” with the examples of comorbidity, stroke, heart disease (e.g., myocardial infarction, angina pectoris), hypertension, diabetes, dyslipidemia, pulmonary tuberculosis, and other diseases including cancer.

Disability status included the presence, severity, and type of disability, including non-disabled, physical disability, encephalopathy, visual impairment, hearing impairment, and others. Disabled persons were registered with the Ministry of Health and Welfare of South Korea; hence, the NHIS data included their disability status. Severity and type of disability were measured according to the Act on Welfare of Persons with Disability.

Smoking was measured with the question: “In your life, have you ever smoked over five packs of cigarettes (100 pieces)?” Drinking was measured using the question, “How many times do you drink a week?”

### 2.3. Statistical analysis

The effects of PA and BMI on infection and mortality associated with COVID-19 were analyzed using logistic regression analysis, which was adjusted for sex, age, insurance type, comorbidities, and region of residence at baseline. Disability and lifestyle (e.g., need for weight management, smoking, and drinking status) were adjusted conjointly. Cases with missing data were excluded from the analysis. The 95% confidence interval (CI) was estimated using the SAS PROC PHREG. Statistical significance was set at *p* < 0.05. Statistical analyses were conducted using the SAS software (SAS Institute Inc., Cary, NC, USA).

## 3. Results

The analysis included 71,488 participants (62% women) over 20 years of age who had received medical checkups from 2015 to 2018. There were 4,363 COVID-19 confirmed cases (6.1%), including 141 deaths (3.3%; 48 women) (Table 1). Among the participants, 418 people with medical aid were developed COVID-19 (12%), with the highest ratio compared to the other quintiles. According to disability status, severe disability was associated with a higher infection rate (13.6%) than mild disability (6.3%) and non-disability (5.9%). People with comorbidities had a higher infection rate (number of comorbidities: 1 = 8%, 2 = 7%, 3 = 10.4%) than those without comorbidities (5.5%).

**Table 1:** Descriptive statistics of COVID-19 infection.

			**All**		**COVID-19 infection**				***p*-value**	**All**		**COVID-19 mortality**				***p*-value**
					**Infected**		**Non-infected**					**Deaths**		**Survivors**		
			***n***	**%**	***n***	**%**	***n***	**%**		***n***	**%**	***n***	**%**	***n***	**%**	
Domain			71,488	100	4,363	6.1	67,125	93.9	<0.0001	4,363	100	141	3.2	4,222	96.8	<0.0001
Characteristic	Sex	Male	27,044	100	1,615	6.0	25,429	94.0	0.2524	1,615	100	93	5.8	1,522	94.2	<0.0001
		Female	44,444	100	2,748	6.2	41,696	93.8		2,748	100	48	1.7	2,700	98.3	
	Age	20–29	6,550	100	281	4.3	6,269	95.7	<0.0001	281	100	0	0.0	281	100.0	<0.0001
		30–39	6,973	100	400	5.7	6,573	94.3		400	100	0	0.0	400	100.0	
		40–49	11,559	100	757	6.5	10,802	93.5		757	100	2	0.3	755	99.7	
		50–59	19,305	100	1,257	6.5	18,048	93.5		1,257	100	10	0.8	1,247	99.2	
		60–69	15,676	100	991	6.3	14,685	93.7		991	100	25	2.5	966	97.5	
		70–79	7,786	100	465	6.0	7,321	94.0		465	100	44	9.5	421	90.5	
		80-	3,639	100	212	5.8	3,427	94.2		212	100	60	28.3	152	71.7	
	Region of residence	Seoul	4,174	100	267	6.4	3,907	93.6	0.9053	267	100	3	1.1	264	98.9	<0.0001
		Daegu	47,057	100	2,871	6.1	44,186	93.9		2,871	100	80	2.8	2,791	97.2	
		Gyeonggi	3,811	100	224	5.9	3,587	94.1		224	100	10	4.5	214	95.5	
		Gyeong-buk	9,339	100	572	6.1	8,767	93.9		572	100	39	6.8	533	93.2	
		others	7,107	100	429	6.0	6,678	94.0		429	100	9	2.1	420	97.9	
	Health insurance premium	Medical aid	3,497	100	418	12.0	3,079	88.0	<0.0001	418	100	18	4.3	400	95.7	0.0253
		1st quintile	12,336	100	846	6.9	11,490	93.1		846	100	19	2.2	827	97.8	
		2nd quintile	10,485	100	588	5.6	9,897	94.4		588	100	11	1.9	577	98.1	
		3rd quintile	12,612	100	741	5.9	11,871	94.1		741	100	23	3.1	718	96.9	
		4th quintile	14,583	100	769	5.3	13,814	94.7		769	100	25	3.3	744	96.7	
		5th quintile	17,975	100	1,001	5.6	16,974	94.4		1,001	100	45	4.5	956	95.5	
Comorbidity	Number of comorbidities	0	53,053	100	2,893	5.5	50,160	94.5	<0.0001	2,893	100	44	1.5	2,849	98.5	<0.0001
		1	8,524	100	684	8.0	7,840	92.0		684	100	29	4.2	655	95.8	
		2	7,169	100	502	7.0	6,667	93.0		502	100	31	6.2	471	93.8	
		3+	2,742	100	284	10.4	2,458	89.6		284	100	37	13.0	247	87.0	
Disability	Presence	Non-disabled	66,859	100	3,974	5.9	62,885	94.1	<0.0001	3,974	100	107	2.7	3,867	97.3	<0.0001
		Disabled	4,629	100	389	8.4	4,240	91.6		389	100	34	8.7	355	91.3	
	Severity	Non-disabled	66,859	100	3,974	5.9	62,885	94.1	<0.0001	3,974	100	107	2.7	3,867	97.3	<0.0001
		Mild	3,317	100	210	6.3	3,107	93.7		179	100	18	10.1	161	89.9	
		Severe	1,312	100	197	13.6	1,133	86.4		210	100	16	7.6	194	92.4	
	Type	Non-disabled	66,859	100	3,974	5.9	62,885	94.1	<0.0001	3,974	100	107	2.7	3,867	97.3	<0.0001
		Physical disability	2,215	100	139	6.3	2,076	93.7		139	100	11	7.9	128	92.1	
		Encephalopathy	372	100	34	9.1	338	90.9		34	100	4	11.8	30	88.2	
		Visual impairment	483	100	26	5.4	457	94.6		26	100	0	0.0	26	100.0	
		Hearing impairment	875	100	69	7.9	806	92.1		69	100	6	8.7	63	91.3	
		Others	684	100	121	17.7	563	82.3		121	100	13	10.7	108	89.3	
Lifestyle	Smoking	No	53,058	100	3,523	6.6	49,535	93.4	<0.0001	4,061	100	130	3.2	3,931	96.8	0.6757
		Yes	18,430	100	840	4.6	17,590	95.4		302	100	11	3.6	291	96.4	
	Drinking	No	60,961	100	4,061	6.7	56,900	93.3	<0.0001	3,523	100	130	3.7	3,393	96.3	0.0005
		yes	10,527	100	302	2.9	10,225	97.1		840	100	11	1.3	829	98.7	
	Weight management	Unnecessary	36,016	100	2,219	6.2	33,797	93.8	0.5137	2,219	100	70	3.2	2,149	96.8	0.7694
		Necessary	35,472	100	2,144	6.0	33,328	94.0		2,144	100	71	3.3	2,073	96.7	
	BMI	Underweight	2,684	100	148	5.5	2,536	94.5	<0.0001	148	100	4	2.7	144	97.3	0.0928
		Normal	27,927	100	1,576	5.6	26,351	94.4		1,576	100	45	2.9	1,531	97.1	
		Overweight	16,784	100	1,061	6.3	15,723	93.7		1,061	100	27	2.5	1,034	97.5	
		Obese	24,093	100	1,578	6.5	22,515	93.5		1,578	100	65	4.1	1,513	95.9	

PA, physical activity; MVPA, moderate-to-vigorous PA; BMI, body mass index.

Of the confirmed cases, 73.75% of the deaths were of participants aged more than 70 years, while 1.41% were of those under 50 years old. Most deaths occurred in people who lived in Daegu (80 people, fatality rate of 2.8%) and Gyeong-Buk (39 people, fatality rate of 6.8%), whereas only 22 people died in other regions. Regarding comorbidities, 44 patients (1.5%) who had no underlying diseases died, while 97 patients (fatality rate, 1 = 4.2%, 2 = 6.2%, over 3 = 13%) died of COVID-19.

### 3.1. Associations between PA and COVID-19

According to logistic regression Model 1 of COVID-19 (Table 2), which was adjusted for characteristics and comorbidity, not engaging in sufficient MVPA (95% CI: 0.989–1.119, *p* = 0.108) did not affect the risk COVID-19. According to logistic regression Model 2 of COVID-19, which was additionally adjusted for the need for weight management, smoking, and drinking status, not engaging in sufficient MVPA (OR: 1.116, 95% CI: 1.046–1.191, *p* < 0.01) predicted a higher risk of COVID-19 than engaging in sufficient MVPA. According to logistic regression Model 3 of COVID-19, which was additionally adjusted for disability status, not engaging in sufficient MVPA (OR: 1.078, 95% CI: 1.014–1.147, *p* < 0.05) still predicted a higher risk of COVID-19 than sufficient MVPA.

**Table 2:** Results of logistic regression analysis according to COVID-19 infection.

**Domain**			**Model 1**				**Model 2**				**Model 3**			
			**Estimate**	**95% CI**		***p*-value**	**Estimate**	**95% CI**		***p*-value**	**Estimate**	**95% CI**		***p*-value**
				**LR**	**UR**			**LR**	**UR**			**LR**	**UR**	
PA		Sufficient MVPA	1	1	1	1	1	1	1	1	1	1	1	1
		Insufficient MVPA	1.052	0.989	1.119	0.1078	1.116	1.046	1.191	0.0009	1.078	1.014	1.147	0.016
BMI		Normal	1	1	1	1	1	1	1	1	1	1	1	1
		Underweight	0.985	0.827	1.173	0.8614	1.009	0.847	1.203	0.9194	0.976	0.82	1.161	0.7817
		Overweight	1.127	1.039	1.223	0.0041	1.128	1.039	1.224	0.004	1.128	1.041	1.223	0.0032
		Obese	1.144	1.062	1.232	0.0004	1.182	1.095	1.277	< 0.0001	1.172	1.09	1.259	<0.0001
Characteristics	Sex	Male	1	1	1	1	1	1	1	1	1	1	1	1
		Female	1.024	0.958	1.094	0.4873	0.754	0.701	0.81	<0.0001	1.038	0.974	1.105	0.2543
	Age	20–29	1	1	1	1	1	1	1	1	1	1	1	1
		30–39	1.334	1.139	1.562	0.0004	1.304	1.113	1.528	0.001	1.358	1.161	1.588	0.0001
		40–49	1.514	1.312	1.746	<0.0001	1.473	1.276	1.7	<0.0001	1.563	1.359	1.799	<0.0001
		50–59	1.426	1.245	1.633	<0.0001	1.332	1.161	1.528	<0.0001	1.554	1.361	1.774	<0.0001
		60–69	1.301	1.131	1.497	0.0002	1.164	1.01	1.342	0.036	1.506	1.314	1.724	<0.0001
		70–79	1.19	1.015	1.395	0.0318	1.008	0.858	1.185	0.9212	1.417	1.217	1.649	<0.0001
		80-	1.11	0.917	1.345	0.2839	0.907	0.747	1.101	0.3225	1.38	1.149	1.657	0.0006
	Region of residence	Seoul	1	1	1	1	1	1	1	1	1	1	1	1
		Daegu	0.905	0.794	1.032	0.1367	0.898	0.788	1.025	0.1113	0.951	0.835	1.082	0.4453
		Gyeonggi	0.906	0.754	1.089	0.2949	0.916	0.761	1.101	0.3489	0.914	0.761	1.098	0.335
		Gyeong-Buk	0.905	0.777	1.054	0.1985	0.894	0.767	1.042	0.1511	0.955	0.822	1.109	0.5451
		others	0.901	0.768	1.055	0.1953	0.898	0.766	1.053	0.1863	0.94	0.803	1.101	0.4424
	Health insurance premium	Medical aid	1	1	1	1	1	1	1	1	1	1	1	1
		1st quintile	0.568	0.502	0.644	<0.0001	0.564	0.498	0.639	<0.0001	0.542	0.479	0.614	<0.0001
		2nd quintile	0.471	0.412	0.538	<0.0001	0.468	0.409	0.535	<0.0001	0.438	0.384	0.499	<0.0001
		3rd quintile	0.494	0.435	0.562	<0.0001	0.491	0.432	0.558	<0.0001	0.46	0.405	0.522	<0.0001
		4th quintile	0.429	0.378	0.486	<0.0001	0.419	0.369	0.475	<0.0001	0.41	0.362	0.465	<0.0001
		5th quintile	0.447	0.396	0.505	<0.0001	0.427	0.378	0.482	<0.0001	0.434	0.385	0.49	<0.0001
Comorbidity	Number of comorbidities	0	1	1	1	1	1	1	1	1	1	1	1	1
		1	1.491	1.365	1.629	<0.0001	1.486	1.36	1.624	<0.0001	1.513	1.387	1.65	<0.0001
		2	1.292	1.169	1.429	<0.0001	1.269	1.148	1.404	<0.0001	1.306	1.183	1.44	<0.0001
		3+	1.971	1.725	2.251	<0.0001	1.941	1.698	2.218	<0.0001	2.003	1.762	2.278	<0.0001
Lifestyle	Smoking	No					1	1	1	1	1	1	1	1
		Yes					0.746	0.686	0.812	<0.0001	0.671	0.622	0.725	<0.0001
	Drinking	No					1	1	1	1	1	1	1	1
		yes					0.38	0.334	0.431	<0.0001	0.414	0.368	0.466	<0.0001
	Weight management	Unnecessary					1	1	1	1	1	1	1	1
		Necessary					0.928	0.867	0.992	0.0289	0.98	0.922	1.042	0.5137
Disability	Presence	Non-disabled									1	1	1	1
		Disabled									1.452	1.302	1.618	<0.0001
	Severity	Non-disabled									1	1	1	1
		Mild									1.07	0.927	1.234	0.3581
		Severe									2.5	2.129	2.936	<0.0001
	Type	Non-disabled									1	1	1	1
		Physical disability									1.06	0.89	1.262	0.5166
		Encephalopathy									1.592	1.117	2.268	0.0101
		Visual impairment									0.9	0.606	1.338	0.6035
		Hearing impairment									1.355	1.057	1.736	0.0164
		Others									3.401	2.787	4.15	<0.0001

PA, physical activity; MVPA, moderate-to-vigorous PA; BMI, body mass index.

### 3.2. Associations between PA and COVID-19-associated mortality

According to logistic regression Model 1 of COVID-19-associated mortality (Table 3), which was adjusted for characteristics and comorbidity, not engaging in sufficient PA (OR: 1.548, 95% CI: 1.051–2.279, *p* < 0.05) predicted a higher risk of COVID-19-associated mortality than engaging in sufficient PA. According to logistic regression Model 2 of COVID-19-associated mortality, which was additionally adjusted for the need for weight management, smoking, and drinking status, not engaging in sufficient MVPA (OR: 1.623, 95% CI: 1.078–2.445, *p* < 0.05) still predicted a higher risk of COVID-19-associated mortality than engaging in sufficient MVPA. According to logistic regression Model 3 of COVID-19-associated mortality, which was additionally adjusted for disability status, engaging in sufficient MVPA (95% CI: 0.96–1.882, *p* = 0.085) did not predict COVID-19-associated mortality. However, the presence of disability and the levels of severity of disability predicted COVID-19-associated mortality.

**Table 3:** Results of logistic regression analysis according to COVID-19 mortality.

**Domain**			**Model 1**				**Model 2**				**Model 3**			
			**Estimate**	**95% CI**		***p*-value**	**Estimate**	**95% CI**		***p*-value**	**Estimate**	**95% CI**		***p*-value**
				**LR**	**UR**			**LR**	**UR**			**LR**	**UR**	
PA		Sufficient MVPA	1	1	1	1	1	1	1	1	1	1	1	1
		Insufficient MVPA	1.548	1.051	2.279	0.0269	1.623	1.078	2.445	0.0204	1.344	0.96	1.882	0.0846
BMI		Normal	1	1	1	1	1	1	1	1	1	1	1	1
		Underweight	0.737	0.224	2.424	0.6159	0.824	0.246	2.752	0.7524	0.945	0.335	2.665	0.9149
		Overweight	0.829	0.487	1.411	0.4894	0.862	0.504	1.473	0.5859	0.888	0.548	1.441	0.6315
		Obese	1.189	0.769	1.839	0.4368	1.336	0.836	2.134	0.2258	1.462	0.993	2.152	0.0544
Characteristic	Sex	Male	1	1	1	1	1	1	1	1	1	1	1	1
		Female	0.272	0.182	0.405	<0.0001	0.231	0.153	0.35	<0.0001	0.291	0.204	0.414	<0.0001
	Age	20-59	1	1	1	1	1	1	1	1	1	1	1	1
		60-69	4.48	2.213	9.067	<0.0001	4.059	2.001	8.231	0.0001	5.786	2.896	11.562	<0.0001
		70-79	19.832	10.125	38.846	<0.0001	18.229	9.24	35.965	<0.0001	23.367	12.241	44.605	<0.0001
		80-	63.703	31.81	127.573	<0.0001	57.833	28.652	116.731	<0.0001	88.256	46.492	167.54	<0.0001
	Region of residence	Seoul	1	1	1	1	1	1	1	1	1	1	1	1
		Daegu	2.165	0.598	7.835	0.2391	2.079	0.564	7.668	0.2715	2.522	0.791	8.042	0.1178
		Gyeonggi	4.091	0.954	17.531	0.0578	3.761	0.858	16.493	0.0791	4.112	1.118	15.13	0.0334
		Gyeong-buk	3.629	0.967	13.623	0.0561	3.301	0.863	12.622	0.0809	6.439	1.972	21.029	0.002
		others	1.749	0.411	7.437	0.4492	1.556	0.359	6.751	0.555	1.886	0.506	7.029	0.3447
	Health insurance premium	Medical aid	1	1	1	1	1	1	1	1	1	1	1	1
		1st quintile	0.425	0.203	0.888	0.0229	0.443	0.212	0.926	0.0304	0.511	0.265	0.983	0.0445
		2nd quintile	0.552	0.239	1.273	0.1633	0.572	0.248	1.321	0.1909	0.424	0.198	0.907	0.0269
		3rd quintile	0.824	0.41	1.66	0.5888	0.829	0.41	1.675	0.6012	0.712	0.38	1.335	0.2894
		4th quintile	0.579	0.289	1.16	0.1233	0.593	0.295	1.192	0.1424	0.747	0.403	1.385	0.3542
		5th quintile	0.554	0.292	1.054	0.072	0.563	0.294	1.079	0.0836	1.046	0.598	1.829	0.8746
Comorbidity	Number of comorbidities	0	1	1	1	1	1	1	1	1	1	1	1	1
		1	1.88	1.117	3.164	0.0174	1.815	1.075	3.064	0.0258	2.867	1.78	4.616	<0.0001
		2	1.893	1.119	3.202	0.0173	1.853	1.093	3.141	0.022	4.262	2.664	6.817	<0.0001
		3+	2.303	1.345	3.946	0.0024	2.347	1.362	4.045	0.0021	9.703	6.15	15.31	<0.0001
Lifestyle	Smoking	No					1	1	1	1	1	1	1	1
		Yes					0.305	0.151	0.614	0.0009	0.346	0.186	0.644	0.0008
	Drinking	No					1	1	1	1	1	1	1	1
		yes					1.5	0.733	3.068	0.267	1.143	0.611	2.139	0.6759
	Weight management	Unnecessary					1	1	1	1	1	1	1	1
		Necessary					0.864	0.564	1.325	0.5035	1.051	0.752	1.471	0.7694
Disability	Presence	Non-disabled									1	1	1	1
		Disabled									3.461	2.318	5.168	<0.0001
	Severity	Non-disabled									1	1	1	1
		Mild									2.981	1.729	5.139	<0.0001
		Severe									4.041	2.394	6.822	<0.0001
	Type	Non-disabled									1	1	1	1
		Physical disability									3.106	1.629	5.92	0.0006
		Encephalopathy									4.819	1.668	13.92	0.0037
		Visual impairment									<0.001	<0.001	>999.999	0.9787
		Hearing impairment									3.442	1.458	8.127	0.0048
		Others									4.35	2.372	7.979	<0.0001

PA, physical activity; MVPA, moderate-to-vigorous PA; BMI, body mass index.

## 4. Discussion

This study aimed to determine the effect of MVPA on COVID-19 and the association between BMI and COVID-19, considering disability status. Investigating NHIS data on COVID-19 revealed several associations. Engaging in insufficient MVPA was associated with higher risk of infection and mortality associated with COVID-19, depending on confounding variables.

The importance of engaging in an active lifestyle was found to be influential on the risk of COVID-19 over the pandemic period. The result of this study aligns with those of a recent study, which reported the decrement of PA engagement over the period of COVID-19 (20) and the lower likelihood of developing COVID-19 (14). However, the current study has taken a step forward by utilizing the most recent data available before the pandemic. It is widely known that engaging in adequate MVPA is associated with positive health outcomes. However, the current study adds another piece of information on the role of engaging in MVPA. For example, a special focus should be paid to individuals with disabilities regardless of the type of disabilities. During the pandemic, disadvantaged populations may experience issues with accessing health information and PA programs in local communities. For example, statistics show a 4% decrement in PA participation by individuals with disabilities in 2021, compared to the participation rate in 2020 (21). Thus, it might be important to dedicate effort toward improving their participation. A recent review emphasized the role of supportive environments to stimulate one's autonomy, competence, and relatedness (i.e., social supports from close people) in PA settings (22). Given that older individuals are more susceptible to infection, these efforts must be focused on them. For example, local communities may provide newly developed programs to educate PA leaders, who may motivate older adults in their communities.

Engaging in insufficient MVPA could play an important role in reducing mortality associated with COVID-19. Previously, insufficient MVPA was reported as a negative predictor of all-cause mortality (23). However, the relation has not been confirmed in the patients with COVID-19. Interestingly, the estimates from the mortality results are higher than from the infection results, which suggests the importance of regular MVPA participation. Since the infection is an on-going phenomenon, engaging in MVPA should be recommended in South Korea. Particular attention should be paid to people, who are aged or physically disabled. It is noted that individuals with disability show much higher risks of mortality than those without disability. Thus, barriers to PA should be eliminated, particularly perceived barriers, such as feeling uncomfortable, a lack of time, and other priorities (24). A technique to overcome these barriers could be providing clear intervention based on the stage of intention of individuals with disability. It is important for researchers and regional practitioners to consider levels of motivation and volition (i.e., psychological willingness to participate in PA) (25) to provide proper PA guidelines.

This study has some limitations. First, this study's sampling period (8 months) was relatively short to determine the tendency of COVID-19, as the situation continues to evolve. At the beginning of this study, in August 2020, there were 4,222 confirmed cases and only 141 people had died of COVID-19 in South Korea. However, there are now more confirmed cases and deaths in South Korea. Second, it is difficult to generalize the results of South Korea and apply them to the rest of the world. Furthermore, the fatality rate in South Korea was deferred from the rate of the world. Thus, the results of this study may not be generalized. Finally, COVID-19 variants continue to evolve and affect individuals differently (26). As a result, the results from this study could not be applied to different variants. Thus, further studies must include various cases and variants to strengthen the associative findings between PA and COVID-19. Lastly, the current study did not examine the roles of light PA and sedentary behavior due to the limited data from the secondary source. Future research may include those variables to clarify the results of the current study.

In conclusion, this study examined the role of sufficient MVPA on both COVID-19 and associated mortality and found that engaging in sufficient MVPA may play a role in reducing the risk of COVID-19 and associated mortality. The main implication of the results is that an active lifestyle should be promoted in the community. In addition, urgent implementation is needed for people who are older or physically disabled.

## Data availability statement

Data can be accessed with the permission of NHIS. Datasets of NHIS can be found here: https://nhiss.nhis.or.kr/bd/ay/bdaya001iv.do.

## Author contributions

Conceptualization and analysis: I-HO and S-YP. Writing: SP and HK. All authors contributed to the article and approved the submitted version.
